# Decoding the Public’s Real-Time Emotional and Cognitive Responses to the Changing Climate on Social Media: Computational Analysis Using Weibo and Meteorological Data

**DOI:** 10.2196/70336

**Published:** 2025-10-03

**Authors:** Yucan Xu, Jiehu Yuan, Sijia Li, Qiuyan Liao

**Affiliations:** 1 School of Public Health LKS Faculty of Medicine University of Hong Kong Hong Kong China (Hong Kong); 2 Department of Social Work and Social Administration Faculty of Social Sciences University of Hong Kong Hong Kong China (Hong Kong)

**Keywords:** climate change, emotional well-being, pro-environmental behaviors, regional vulnerability, cognitive responses, psycholinguistic analysis, Weibo, infodemiology

## Abstract

**Background:**

Climate change poses a significant threat to mental health and well-being worldwide. Existing research on the associations between climate change–related events and mental well-being primarily focuses on clinical outcomes and often measures associations at single time points. The long-term effects and variability of the changing climate on more subtle nonclinical but widespread mental well-being remain relatively unexplored. Additionally, the underlying mechanisms that link changing climate events to real-time emotional well-being and pro-environmental actions have rarely been studied. Revealing real-time nonclinical mental well-being and its underlying mechanism is crucial for the early detection of at-risk individuals. This knowledge can also inform future interventions aimed at improving the public’s risk perception and empowering communities to manage related challenges effectively.

**Objective:**

This study aimed to understand the association between the changing climate and expressed emotional well-being by integrating multiple data sources, including social media posts about climate change on Weibo (N=76,514), 20 years of regional meteorological data (N=216,476 records), and regional vulnerability data in China.

**Methods:**

This study proposed and tested a new mechanism that connects meteorological factors with expressed emotional well-being through three cognitive responses identified from social media posts: thinking styles, social affiliations, and somatosensory experiences. Psycholinguistic analysis, structural equation modeling (SEM), and multiple regression models were used to examine the mediation of these three conceptual factors, as well as the moderating effects of regional vulnerability and seasonal changes on the influence of climate change on the public’s expressed emotional well-being and downstream pro-environmental tendencies.

**Results:**

The SEM results revealed that extreme hot days are associated with decreased emotional well-being when talking about climate change (total effect=–0.712, 95% CI –0.894 to –0.531, P<.001), and these effects were mediated by three proposed mediators: social affiliations (indirect effect=–0.445, 95% CI –0.537 to –0.347, P<.001), analytical-intuitive thinking style (indirect effect=–0.100, 95% CI –0.126 to –0.073, P<.001), and somatosensory experiences (indirect effect=0.022, 95% CI 0.005-0.041, P=.02). Additionally, regression analysis indicated that the association between increased temperatures and expressed emotional well-being is moderated by seasonal changes (β=–.091, 95% CI –0.159 to –0.023, P=.009) and regional population density (β=–.068, 95% CI –0.118 to –0.018, P=.007). In the crude model examining associations between weather indices and expressed pro-environmental tendencies, the results showed that extreme hot days are associated with reduced pro-environmental tendencies (odds ratio [OR]=0.802, 95% CI 0.747-0.861, P<.001). However, after controlling for expressed emotional well-being and cognitive responses, such associations were less pronounced.

**Conclusions:**

The findings highlight the need for interventions that promote mental well-being in response to climate change and the importance of cognitive responses in developing positive coping strategies and enhancing emotional resilience. This approach could empower individuals to create a positive self-reinforcing cycle that encourages pro-environmental behaviors.

## Introduction

### Background

Climate change is a significant threat to mental health and well-being worldwide [1]. Subacute or long-term climate change–related events, such as heatwaves and higher temperatures, have been found to be associated with increased mental health issues and even suicide rates [2]. For example, a large sampled study examining the relationship of historical meteorological data and information of two million US residents found that shifting of monthly temperatures between 25 °C and 30 °C or above increased the probability of mental health difficulties by 0.5% points, and 1°C of 5-year warming was associated with a 2% point increase in the prevalence of mental health issues [3]. Measuring and monitoring the impacts of climate change on mental health requires a long-term and pragmatic approach by incorporating multiple data sources. Unfortunately, most studies to date investigating the impact of climate change on mental well-being have been conducted with cross-sectional surveys, which may suffer from recall biases or fail to evaluate the underlying mechanisms [4]. In addition, they have focused solely on clinical mental health outcomes, such as hospital attendance or admission, and suicide. Little is known about the real-time emotional impacts, such as negative moods, and the more subtle but widespread impact on expressed mental well-being related to climate change. The more subtle mental well-being impact can serve as an early screening signal to identify people who are at risk for clinical mental health issues and inform future interventions, which informs communication to promote public awareness of risk and strategies to empower the public to cope with related risks. There is also insufficient understanding of how climate change–induced emotional well-being influences downstream pro-environmental behaviors. To answer those inquiries and address these gaps, this research aimed to link social media data and meteorological data to provide a more comprehensive understanding of the impact of climate change on the public’s emotional well-being, the potential mechanisms through cognitive responses, and their impact on pro-environmental tendencies.

### Climate Change and Emotional Well-Being

There has been extensive evidence that climate change–related events are associated with adverse mental health outcomes, including various mental disorders, hospitalizations due to mental health, and even suicide [4,5]. Both more chronic meteorological changes and acute extreme weather pose significant threats to individuals’ psychological well-being. Concerning the impacts of long-term meteorological changes, a systematic review suggested most meteorological factors, such as temperature increase, aridity, and heatwaves, are associated with mental health outcomes, including hospital admissions, exacerbation of mental health conditions, sleeping difficulty, and suicide [6]. It has been consistently reported that acute extreme weather events, such as flooding and drought, are associated with diverse mental health issues, including posttraumatic stress disorder, anxiety, and depression [7]. Previous studies have examined the links between weather indicators and mental health outcomes, but many of them have focused primarily on posttraumatic scenarios, such as heat waves [8]. This has resulted in a lack of attention to nonacute meteorological changes, such as gradual temperature shifts. It is posited that the impact of different meteorological events, acute and nonacute, may vary and is worth further investigation. As for the outcomes, existing studies on the impact of climate change on mental health outcomes mainly focus on clinical outcomes, including suicides, hospitalizations for mental illness, and severity of symptoms [4]. Except for clinical outcomes, various nonclinical outcomes, such as mood, positive and negative affect, and overall well-being, are also used to assess the impact of climate change on mental health [4]. Although those measures may not directly indicate the clinical profile of individuals, they allow for a broader understanding of the effects of climate change on mental health in wider populations. This broader generalizability can be beneficial for the early detection of individuals who are at risk.

### Cognitive Responses as Potential Mediators

Although climate change–related events can directly affect individuals’ mental well-being [5], the literature has documented several important mechanisms that could explain how climate change can indirectly impair human mental health. First, the physiological mechanism argues that exposure to heat waves may induce biological changes in neural mechanisms that may further affect and exacerbate symptoms in individuals with mental health conditions [9]. Second, cognitive perspectives have found that sleep deprivation caused by environmental changes can affect human cognitive capacity and contribute to mental health problems [10]. Third, the environmental and social process states that climate change–related events can erode social and community well-being by reducing economic and agricultural outputs, ultimately leading to mental health problems [11]. However, there remains limited knowledge regarding how cognitive responses, such as individuals’ thinking styles, affiliative strategies, and personal somatosensory experiences, interact with climate change–related events and mental well-being. Investigating these cognitive strategies can offer important insights into mitigating the impacts of climate change on mental well-being.

#### Thinking Style

In accordance with the Stimulus-Organism-Response framework, meteorological changes in the environment can induce cognitive and emotional responses in human beings and ultimately impact human health directly or indirectly [12]. According to Daniel Kahneman, the cognitive responses to a stimulus or information involve two styles: intuitive thinking (system 1) and analytical thinking (system 2) [13]. Intuitive thinking is a process that is usually fast, automatic, and sometimes unconscious, while analytical thinking is a process that is more logical and deliberative. People are usually predisposed to a specific cognitive style, the more intuitive or analytic one [14]. Individuals who adopt an analytical thinking style tend to perceive more control in a specific situation compared to those who mainly adopt the intuitive thinking style [15,16]. Conversely, intuitive thinking is associated with greater skepticism toward climate change [17]. Nevertheless, these studies have not examined thinking styles as a potential mediator of the impacts of the changing climate on emotional well-being.

#### Social Connectedness and Affiliations

Based on social safety theory, social connectedness and affiliations (perceived as being connected and affiliated with others) serve as an important factor that buffers the emotional impacts of major environmental hazards [18]. Extensive research has demonstrated that individuals with high affiliative motives are more likely to experience positive emotions and exhibit better mental health outcomes compared to those with self-focused motives [19,20]. Engaging in affiliate motives and altruistic acts could alleviate and help prevent mental health difficulties, leading to better emotional outcomes [21]. In turn, those positive emotions encourage individuals to help others who are in need and form an ongoing cooperative relationship [22]. When faced with climate change events with low-dread risks, such as heat waves, individuals tend to prioritize individual adaptation strategies, such as seeking shade, decreasing clothing, or using air-conditioning, rather than engaging in the more costly pro-environmental behaviors [23]. A recent review on drivers for climate action revealed that social groups and the individual attachment to these groups can be influential on individual climate actions [24]. However, there is limited knowledge regarding the triangle relationship between affiliate motives, changing temperatures and heat waves, and the public’s emotional well-being.

#### Somatosensory Experiences

The existing literature elucidates the mechanism linking somatosensory, brain, and affect systems, revealing the pathway of how somatosensory systems mediate the impact of climate change on mental health [10,25]. The process begins with the body’s sensory system receiving signals from climate change–related stimuli and subsequently transmitting those signals to the brain systems that control affective function. The somatosensory system is responsible for the conscious perception of various bodily somatic sensations, including touch, pressure, pain, temperature, body position, movement, and vibration [26]. Previous research on the association between different body sensations and emotions has demonstrated that somatosensory experiences could trigger conscious emotional responses and that different bodily sensations are associated with different emotions [27]. Given the connection between somatosensory experiences and emotional responses, it is reasonable to speculate that individuals may perceive and interpret different bodily signals when exposed to increased temperatures and extreme weather events, potentially leading to the activation of negative emotional states. However, empirical testing is required to confirm this hypothesis.

### Emotional Well-Being and Pro-Environmental Tendencies

The cognitive and emotional impacts of climate change can go beyond and influence people’s behaviors. Research indicates that negative emotions triggered by climate change can have a bidirectional impact on pro-environmental behaviors [28]. On the one hand, coping mechanisms suggest that negative emotions triggered by climate change–related events can motivate individuals to take action to mitigate the negative impacts of climate change [29]. On the other hand, some argue that chronic negative mental health problems may lead to eco-paralysis, a state in which individuals passively respond to climatic and ecological challenges, feeling hopeless and powerless [30]. A recent study also reported an association between higher climate anxiety and fewer pro-environmental behaviors, highlighting that climate anxiety leads to lower efficacy and dampens individuals’ ability to take climate actions [31]. Despite some evidence, existing studies have yet to examine whether the more real-time negative impact of climate change on emotional well-being would harm individuals’ pro-environmental intentions and tendencies.

### Social Media Data and Climate Change

Numerous studies have demonstrated social media’s potential to understand the public’s perception and emotional responses to climate change–related events [32-34]. Through the use of natural language processing and network analysis, researchers have been able to study public reactions and sentiments to climate policy actions on social media platforms, such as X (formerly known as Twitter) [35]. Previous studies have demonstrated that linguistic expressions, including content features (eg, words and context), style features (eg, punctuations and emoji), and word categories (eg, discrete emotion dictionaries), have shown strong predicting power for various mental well-being outcomes, including depression and suicide [36,37]. Sentiment and emotional distress identified from such digital content can be used for early detection of individuals who are in need and provide in-time interventions [38]. Among those psycholinguistic methods, the *Linguistic Inquiry and Word Count* (LIWC) dictionary has demonstrated great potential and validity in capturing social media users’ emotional and cognitive responses to science communication agendas, such as climate change [39,40] and the COVID-19 pandemic [41,42].

Although social media data has been widely used to comprehend public reactions to climate change, only a limited number of studies have used a combination of social media data and offline meteorological data to monitor the influence of offline temperature changes and extreme weather events on the public’s emotional responses to climate change [43,44]. This approach may provide a more comprehensive picture of real-time interactions between climate change and the public’s affective responses in a large-scale population.

### Study Aims

Building on the knowledge gaps discussed earlier, this study aimed to investigate the real-time associations between acute extreme meteorological events, including extreme daily temperatures and precipitations; more long-term meteorological changes, including deviations in daily temperatures and precipitations; and the public’s expressed emotional well-being and cognitive responses when discussing related concepts using one popular online social media platform: Weibo data and offline regional meteorological and vulnerability data. Guided by the climate response–related theories as our theoretical foundation, we proposed and tested one novel mechanism connecting meteorological indicators and emotional responses through three cognitive pathways. Specifically, our hypotheses are:

- Hypothesis (H)1: Extreme meteorological events and meteorological changes are associated with the public’s emotional well-being, indicated by climate change discourse. Specifically, extreme hot days and increased temperatures are associated with more negative emotional well-being.
- H2: The association of H1 varies by region and time. People who live in regions with higher vulnerability and in seasons with hotter days (eg, summer) are more prone to be influenced.
- H3: The association of H1 is mediated by individuals’ intuitive-analytical thinking style, social affiliations, and somatosensory experiences. Specifically, extreme hot days and hotter temperature changes are associated with an intuitive-leaning thinking style, a less affiliative attitude, and a higher level of somatosensory experience, which are further associated with more negative emotional well-being.
- H4: Extreme hot days have a negative impact on people’s pro-environmental tendencies. This effect is channeled through their emotional well-being, where extreme hot days are associated with poorer emotional well-being and hence a lower willingness to be pro-environmental.

## Methods

### Dataset

Three parts of data were collected to answer the research questions and were available publicly: First, meteorological data were obtained to identify and measure extreme meteorological events and changes. Second, social media data from Weibo were gathered to gauge the public’s cognitive responses and emotional well-being, as well as their inclinations to be pro-environmental. Finally, regional vulnerability data were collected to ascertain regional variations. Details of the three data collection processes are reported in the *Data Collection* section in Multimedia Appendix 1.

### Study Measures

#### Extreme Weather Indicators and Temperature Changes

There are two common approaches to defining extreme weather. The first approach uses a constant value to set the threshold. Various temperature thresholds, ranging from 25 °C to higher than 40 °C, have been used to define extreme heat indices [45]. For example, one study used 25°C to indicate hot days [46], while the National Weather Service in the United States adopts a threshold of 40.6°C in the daytime and greater than or equal to 26.7°C in the nighttime for its excessive heat watch and warning system [47]. Additionally, another study considered a heat wave as the maximum temperature at a station reaching at least 40 °C for the plains and at least 30 °C for hilly regions [48]. The second approach is to choose a relative threshold that is varied by the empirical situation of the location [49]. For instance, using the 90th percentile as the cutoff for extreme weather indices ensures that 10% of events are classified as “extreme.” This method is beneficial and more robust because it considers the geographical context and relative heat adaptations. We selected the 10th and 90th percentiles as cutoff points for defining extreme weather indices in line with the recommendations by Stephenson et al [49]. We recognized that using the 5th and 95th percentiles might yield different results. However, choosing more extreme values as cutoffs could result in fewer data points, which would limit the interpretability of the data. Based on this approach, we created four indicators: extreme hot days, extreme cold days, temperature changes, and precipitation changes. The detailed method is reported in the *Weather Indicators* section in Multimedia Appendix 1.

#### Outcomes

##### Expressed Emotional Well-Being

Emotional well-being refers to an individual’s overall emotional state, encompassing how positively one feels about their life in general [50]. Consequently, emotional well-being expressed regarding climate change captures the feelings and emotional states that arise when facing climate change. Expressed emotional well-being was the primary outcome identified through the analysis of psycholinguistic cues in individuals’ Weibo posts. These cues were evaluated using the affect categories of the Simplified Chinese version of the *Linguistic Inquiry and Word Count* (SC-LIWC) dictionary, which is a validated adaptation of the original LIWC dictionary [51]. The affect categories encompass positive and negative sentiments, as well as three discrete negative emotions: anger, anxiety, and sadness. Following Monzani et al’s [41] method, we created an emotional well-being index by subtracting the negative sentiment score from the positive sentiment score to indicate the overall emotional well-being expressed for a post. A higher emotional well-being index indicates a more positive emotional well-being loading. Instead of measuring each user’s long-term subjective well-being [52], we assessed expressed emotional well-being at the post level. The goal is to understand the relationships between extreme weather indices and expressed emotional well-being in the context of relevant weather events. By limiting our analysis to posts specifically related to climate change, we have ensured that the discussions are directly tied to climate change issues. In contrast, focusing on all posts from each user may introduce confounding factors, as their emotional well-being could be influenced by unrelated topics (eg, COVID-19) and daily life events (eg, relationship problems) rather than the weather indices.

##### Pro-Environmental Tendency

The pro-environmental tendency of Weibo users was a secondary outcome evaluated using a binary classification of their posts. The keywords and phrases used for the classification were primarily based on the Pro-Environmental Behavior questionnaire (PEB) curated and validated by Kalamas et al [53], covering 50 PEB items in 7 categories: saving energy, consumption, activism, recycling, transportation, diets, and waste management. The full keyword list can be found in Table S1 in Multimedia Appendix 1.

##### Cognitive Responses

To assess the cognitive responses of the public, posts from individual accounts were analyzed using the LIWC dictionary. The analysis focused on three proposed cognitive responses: analytical-intuitive thinking style, social affiliations, and somatosensory experiences. The detailed information for measuring three cognitive responses is reported in the *Measuring Cognitive Responses* section in Multimedia Appendix 1.

##### Regional Vulnerability Indicators

Followed by a national study of regional health vulnerability to extreme heat in China [54] and the framework of vulnerability [55], three indicators were used to measure regional vulnerability: exposure, sensitivity, and adaptability. A detailed description of the three vulnerability indicators is reported in the *Vulnerability Indicators* section in Multimedia Appendix 1.

### Statistical Analysis

Overall, this study applied computational social science methods to facilitate the understanding of the emotional and behavioral impact of the changing climate [56]. Psycholinguistic analysis for climate change–related Weibo posts was conducted with the SC-LIWC dictionary. Descriptive analysis was conducted to describe the initial associations between meteorological indicators and the public’s emotional and cognitive responses with mixed correlations. Next, structural equation modeling (SEM) was used to analyze the mediating effect of the intuitive-analytical thinking style, social affiliations, and somatosensory experiences on the associations between meteorological indicators and emotional well-being. SE values were generated through 1000 bootstrapping. The 95% CI was calculated using the adjusted bootstrap percentile (BCa) method, which is the bias-corrected method for CI estimation [57]. Fit indices were used to evaluate the model fit, including the comparative fit index (CFI>0.90), the goodness-of-fit index (GFI>0.90), and the root mean square error of approximation (RMSEA<0.08) [58,59]. Model modification indices were used to indicate the potential correlations between mediators. Multivariate linear regressions were used to detect the moderation and interaction effect of regional vulnerability factors. Multiple logistic regression models were used to estimate the power of environmental indicators in predicting pro-environmental tendencies when controlling different confounders. The variance inflation factor (VIF) was used to detect multicollinearity, where variables with VIF>5 were removed from selected models [60]. SEM was conducted with Mplus (version 8.3), and all other analyses were performed with the Python *statsmodel* package (version 0.13.1).

#### Cross-Dataset Merge

We initially combined meteorological data from the National Oceanic and Atmospheric Administration (NOAA) with vulnerability data using the IP address at the provincial level. Next, we aligned these combined environmental data with social media data by matching the corresponding date and province (as indicated by the IP address) of each post.

### Ethical Considerations

Data were used complying with ethical guidelines for internet research [61]. All data are publicly open access data, and all analyses were performed on public and anonymized metadata; no institutional review board approval was required for the use of these datasets or the completion of this study.

## Results

### Correlations Between Meteorological Factors, People’s Emotional Responses, and Cognitive Processing

**Figure 1:**
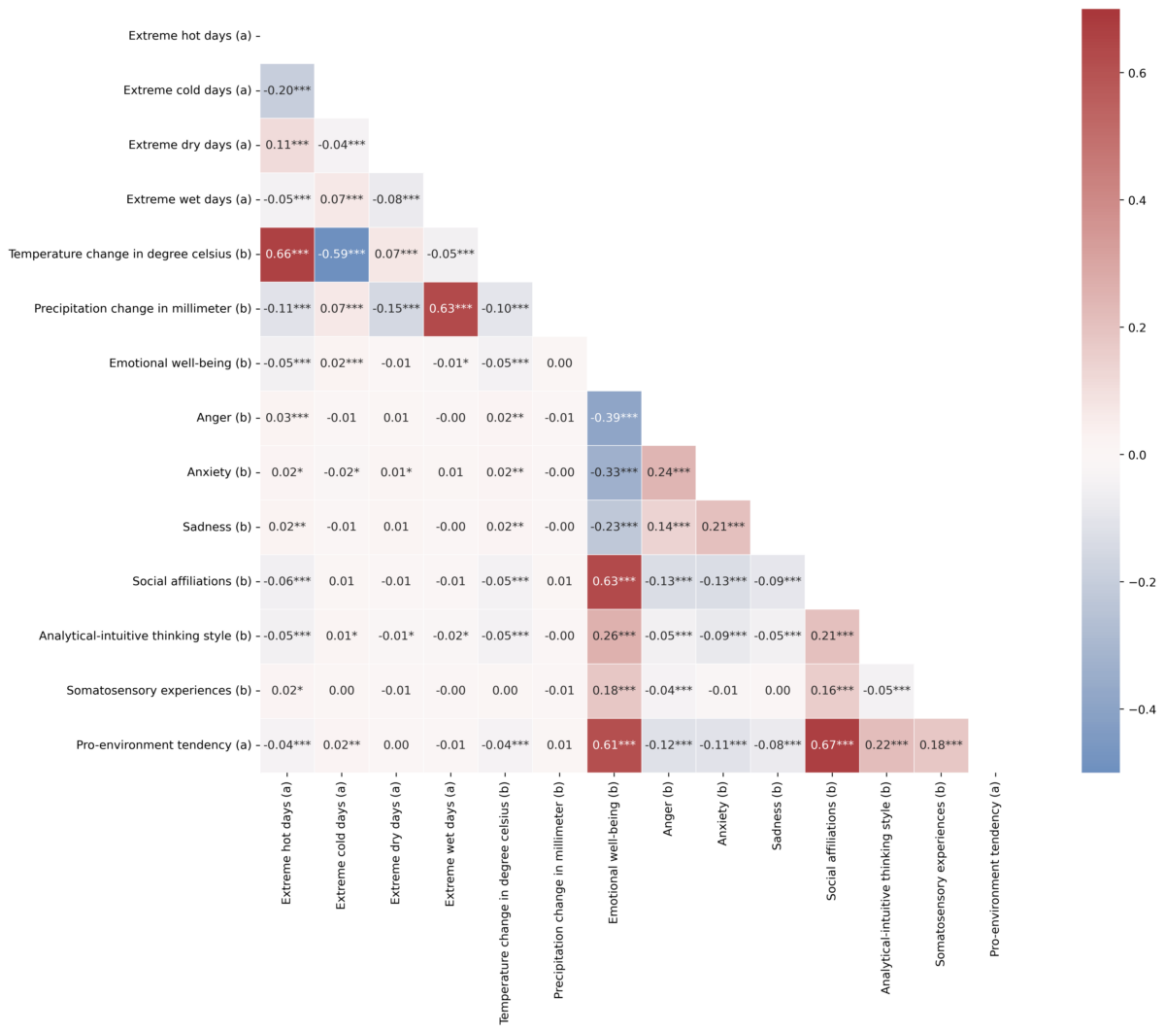
Mixed correlations between meteorological indicators, emotional responses, and cognitive responses for climate change–related posts (N=24,156). (a) Binary variables and (b) continuous variables. Correlations between two continuous variables were evaluated with Pearson correlation, correlations between one continuous variable and one binary variable were evaluated with point-biserial correlation, and correlations between two binary variables were evaluated with Matthews correlation. The red color indicates positive correlations, while the blue color indicates negative correlations. The darker the color, the stronger the correlation. *P<.05, **P<.01, and ***P<.001. The statistical significance (*P* values) presented was adjusted using false discovery rate (FDR) corrections.

Figure 1 visualizes the correlations between environmental meteorological factors and interested measures. Results highlighted that extreme hot days are correlated with more negative emotional well-being expressed in climate change–related posts by Weibo individual users, while extreme cold days are correlated with more positive emotional well-being. Additionally, as the temperature increased, more negative emotional well-being was observed in related posts. Relatedly, extreme hot days and hotter temperatures were correlated with higher scores of anxiety, anger, and sadness (H1 supported). Extreme hot days seemed to have an impact on one’s cognitive processing and behavioral tendency. Specifically, extreme hot days were correlated with an intuitive-leaning thinking style, a greater level of somatosensory experience but reduced affiliation, and a lower inclination toward pro-environmental behaviors. As expected, social affiliations, thinking styles, somatosensory experiences, and emotional well-being were significantly intercorrelated. Individuals who exhibited a higher level of affiliation, an analytical-leaning thinking style, and somatosensory experiences tended to display more positive emotional well-being. Similar results were observed in the outcome of pro-environmental tendencies, where more positive emotional well-being, social affiliations, an analytical-leaning style, and somatosensory experiences were correlated with a greater inclination toward pro-environmental behaviors. On the contrary, negative emotions, including anger, anxiety, and sadness, were negatively associated with pro-environmental tendencies.

### Climate Change–Related Expressed Emotional Well-Being by Season

After controlling for seasons, the presence of extreme hot days remained a robust predictor of emotional well-being (Tables 1 and 2). Specifically, extreme heat or hotter temperature were associated with more negative emotional well-being. Additionally, the results revealed an interaction effect between seasons and temperature changes. Compared to spring, temperature increases in the summer were associated with a significantly higher level of negative emotional well-being (β=–.091, 95% CI –0.159 to –0.023, *P*=.009), as shown in Figure S1 in Multimedia Appendix 1.

**Table 1:** Moderations of seasons to determine the relationship between extreme hot days and emotional well-being (N=24,156).

Variables		β (95% CI)	z Value	*P* value
Extreme hot days (binary)		–0.581 (–0.957 to –0.205)	–3.029	.002^a^
**Seasons**				
	Spring (reference group)	—^b^	—	—
	Summer	–0.866 (–1.083 to –0.649)	–7.817	<.001^a^
	Autumn	–1.152 (–1.407 to –0.897)	–8.859	<.001^a^
	Winter	–0.570 (–0.830 to –0.309)	–4.287	<.001^a^
Summer×extreme hot days		–0.135 (–0.580 to 0.311)	–0.593	.55
Autumn×extreme hot days		0.289 (–0.260 to 0.838)	1.032	.30
Winter×extreme hot days		–0.231 (–0.933 to 0.472)	–0.644	.52

^a^Significant *P* values.

^b^Not applicable.

**Table 2:** Moderations of seasons to determine the relationship between temperature changes and emotional well-being (N=24,156).

Variables			β (95% CI)		z Value		*P* value
Temperature changes (°C)			–0.085 (–0.129 to –0.042)		–3.875		<.001^a^
**Seasons**							
	Spring (reference group)	—^b^		—		—	
	Summer	–0.800 (–1.000 to –0.601)		–7.846		<.001^a^	
	Autumn	–1.125 (–1.355 to –0.894)		–9.567		<.001^a^	
	Winter	–0.581 (–0.822 to –0.340)		–4.724		<.001^a^	
Summer×temperature changes			–0.091 (–0.159 to –0.023)		–2.625		.009^a^
Autumn×temperature changes			0.067 (–0.002 to 0.135)		1.898		.06
Winter×temperature changes			0.044 (–0.025 to 0.112)		1.240		.22

^a^Significant *P* values.

^b^Not applicable.

### Climate Change–Related Expressed Emotional Well-Being by Regional Vulnerability

After controlling for different population vulnerability indexes, the impact of extreme hot days and temperature changes remained significantly associated with emotional well-being (Tables 3 and 4). For vulnerability factors, population density was identified as a significant factor in explaining emotional well-being in both models, controlling for the presence of extreme hot days and hotter temperatures. A higher population density was associated with more negative emotional well-being (β_model 1_=–.256, 95% CI –0.427 to –0.084, *P*=.003; β_model 1s_=–.191, 95% CI –0.345 to –0.038, *P*=.02). The exposure index was significantly associated with the expressed emotional well-being in the model with temperature changes, where a higher exposure index was associated with more negative emotional well-being (β_model 2s_=–.186, 95% CI –0.342 to –0.030, *P*=.02). A follow-up moderation analysis, incorporating interactive terms between expressed emotional well-being and population vulnerability indexes, identified the effect of a significant interaction between population density and temperature change on emotional well-being (β=–.068, 95% CI –0.118 to –0.018, *P*=.007), as shown in Figure S2 in Multimedia Appendix 1. Specifically, on hotter days (mean + 1SD), regions with a higher population density were associated with more negative emotional well-being than regions with a lower population density, while on colder days (mean – 1SD), regions with a higher population density displayed higher emotional well-being than regions with a lower population density (H2 supported).

**Table 3:** Moderations of regional vulnerability indicators to determine the relationship between extreme hot days and emotional well-being (N=24,156).

Variables		β (95% CI)^a^	z Value	*P* value
**Model 1 with population density^b^**				
	Extreme hot days (1)	–0.730 (–0.986 to –0.474)	–5.585	<.001^c^
	Population density, high vs low (2)	–0.256 (–0.427 to –0.084)	–2.920	.003^c^
	(1)×(2)	0.025 (–0.316 to 0.367)	0.144	.89
**Model 2 with exposure index^d^**				
	Extreme hot days (1)	–0.533 (–0.816 to –0.250)	–3.690	<.001^c^
	Exposure index, high vs low (3)	–0.084 (–0.258 to 0.089)	–0.952	.34
	(1)×(3)	–0.268 (–0.622 to 0.085)	–1.487	.14
**Model 3 with sensitivity index^d^**				
	Extreme hot days (1)	–0.823 (–1.051 to –0.595)	–7.065	<.001^c^
	Sensitivity index, high vs low (4)	–0.032 (–0.202 to 0.139)	–0.366	.72
	(1)×(4)	0.247 (–0.093 to 0.588)	1.424	.16
**Model 4 with adaptability index^d^**				
	Extreme hot days (1)	–0.880 (–1.208 to –0.552)	–5.263	<.001^c^
	Adaptability index, high vs low (5)	–0.171 (–0.355 to 0.012)	–1.830	.07
	(1)×(5)	0.239 (–0.144 to 0.622)	1.224	.22

^a^The coefficients (β) and their 95% CIs were estimated using generalized linear regression.

^b^Population density data for each province were extracted from the National Bureau of Statistics. A higher population density indicates that the region’s population density is higher than the median of all provinces.

^c^Significant *P* values.

^d^Regional-level exposure, sensitivity, and adaptability indexes have been calculated and validated by Zhang et al [54].

**Table 4:** Moderations of regional vulnerability indicators to determine the relationship between temperature changes and emotional well-being (N=24,156).

Variables			β (95% CI)^a^		z Value		*P* value
**Model 1s with population density^b^**							
	Temperature changes (1)	–0.061 (–0.097 to –0.024)		–3.261		<.001^c^	
	Population density, high vs low (2)	–0.191 (–0.345 to –0.038)		–2.438		.02^c^	
	(1)×(2)	–0.068 (–0.118 to –0.018)		–2.687		.007^c^	
**Model 2s with exposure index^d^**							
	Temperature changes (1)	–0.119 (–0.163 to –0.075)		–5.283		<.001^c^	
	Exposure index, high vs low (3)	–0.186 (–0.342 to –0.030)		–2.333		.02^c^	
	(1)×(3)	0.033 (–0.020 to 0.087)		1.223		.22	
**Model 3s with sensitivity index^d^**							
	Temperature changes (1)	–0.083 (–0.115 to –0.051)		–5.129		<.001^c^	
	Sensitivity index, high vs low (4)	0.046 (–0.107 to 0.199)		0.594		.55	
	(1)×(4)	–0.035 (–0.085 to 0.016)		–1.342		.18	
**Model 4s with adaptability index^d^**							
	Temperature changes (1)	–0.090 (–0.133 to –0.047)		–4.109		<.001 ^a^	
	Adaptability index, high vs low (5)	–0.123 (–0.288 to 0.042)		–1.461		.14	
	(1)×(5)	–0.009 (–0.062 to 0.044)		–0.333		.74	

^a^The coefficients (β) and their 95% CIs were estimated using generalized linear regression.

^b^Population density data for each province were extracted from the National Bureau of Statistics. A higher population density indicates that the region’s population density is higher than the median of all provinces.

^c^Significant *P* values.

^d^Regional-level exposure, sensitivity, and adaptability indexes have been calculated and validated by Zhang et al [54].

### Mediation of Three Cognitive Responses

**Figure 2:**
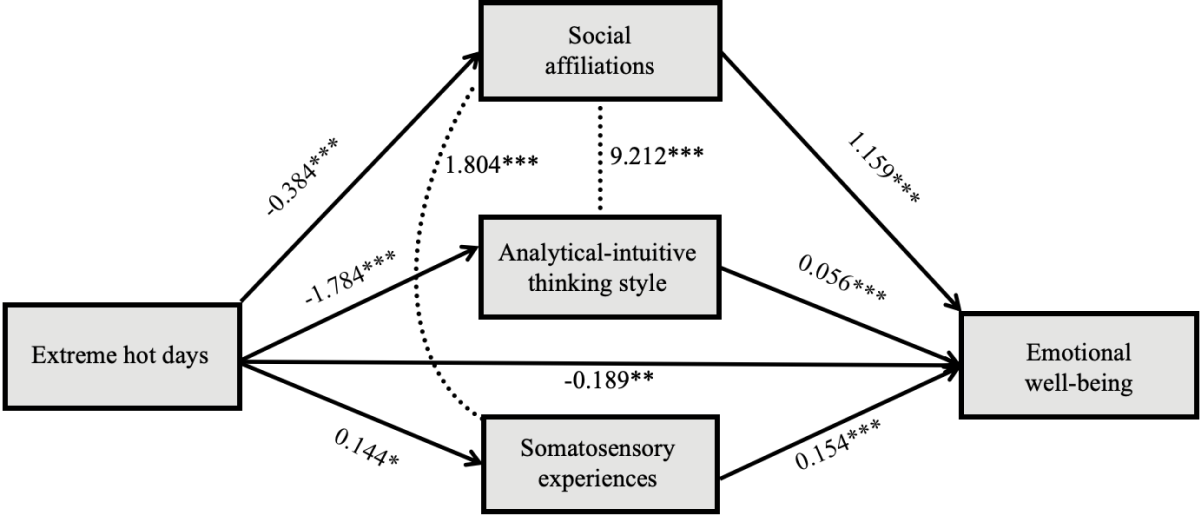
Mediation paths from SEM. Fit indices: CFI=0.997, GFI=0.999, and RMSEA=0.046. The number on each arrow indicates the association between two variables in the model. *P<.05, **P<.01, and ***P<.001. CFI: comparative fit index; GFI: goodness-of-fit index; RMSEA: root mean square error of approximation; SEM: structural equation modeling.

The mediation analysis using SEM, as depicted in Figure 2, confirmed the mediation of the three proposed conceptual factors in the relationship between extreme hot days and emotional well-being. This mediation model accounted for the covariance between social affiliations and thinking styles, as well as the covariance between social affiliations and somatosensory experiences, suggested by model modification indices in Mplus. The results identified a direct effect of extreme hot days on emotional well-being (direct effect=–0.189, 95% CI –0.319 to –0.050, *P*=.007). The indirect effect of social affiliations ranked at the top (indirect effect=–0.445, 95% CI –0.537 to –0.347, *P*<.001), followed by the analytical-intuitive thinking style (indirect effect=–0.100, 95% CI –0.126 to –0.073, *P*<.001) and somatosensory experiences (indirect effect=0.022, 95% CI 0.005-0.041, *P*=.02). Specifically, SEM showed that when there was extreme heat, people showed fewer social affiliations, expressed more bodily sensation experiences, and deployed a more intuitive thinking style about climate change. However, although fewer social affiliations and a more intuitive-leaning thinking style were associated with lower emotional well-being, more somatosensory experiences were positively associated with emotional well-being (H3 partially supported).

We also modeled the mediation pathways between temperature changes and emotional well-being. The model explanation power with temperature changes was lower than the model with extreme hot days (direct effect=–0.030, 95% CI –0.050 to –0.012, *P*=.002; total indirect effect=–0.067, 95% CI –0.082 to –0.050, *P*<.001), as shown in Figure S3 in Multimedia Appendix 1.

### Impacts of Climate Change on a Pro-Environmental Tendency

When no controls were considered (model 1 in Table 5), extreme hot days were associated with a decreased pro-environmental tendency (odds ratio [OR] 0.802, 95% CI 0.747-0.861, *P*<.001). After controlling for emotional well-being (model 2 in Table 6), the impact of extreme hot days weakened (OR 0.903, 95% CI 0.822-0.994, *P*=.04; H4 supported). When controlling for the three additional cognitive factors (model 3 in Table 7), the impact of extreme hot days disappeared. All three cognitive responses remained positively associated with a pro-environmental tendency, among which, social affiliation was the most prominent predictor (OR 1.432, 95% CI 1.409-1.456, *P*<.001). A more analytical-leaning thinking style (OR 1.018, 95% CI 1.015-1.022) and somatosensory experiences (OR 1.038, 95% CI 1.025-1.051) were marginally associated with a higher chance of acting pro-environmentally. When adding regional vulnerability factors into the model (model 4 in Table 8), the results for extreme hot days, emotional responses, and cognitive responses remained as robust as those for model 3. Additionally, high sensitivity was associated with a lower probability of acting pro-environmentally (OR 0.756, 95% CI 0.685-0.835, *P*<.001), while high adaptability was associated with a higher chance of being pro-environment (OR 1.254, 95% CI 1.130-1.393).

**Table 5:** Logistic regression estimates for variables in model 1 for predicting a pro-environmental tendency^a^ (N=24,156).

Variables			Model 1 (crude model) estimates				
			OR^b^ (95% CI)		*P* value		PseudoR2
**Meteorological indicators**							
	Extreme hot vs not extreme hot	0.802 (0.747-0.861)		<.001		0.002	
**Emotional responses**							
	Emotional well-being	—^c^		—		—	
**Cognitive responses**							
	Social affiliations	—		—		—	
	Analytical-intuitive thinking style	—		—		—	
	Somatosensory experiences	—		—		—	
**Regional vulnerability factors**							
	Population density, high vs low	—		—		—	
	Exposure index, high vs low	—		—		—	
	Sensitivity index, high vs low	—		—		—	
	Adaptability index, high vs low	—		—		—	

^a^The pro-environmental tendency is a binary outcome. If a post includes keywords and phrases from any of the seven pro-environmental categories curated and validated by Kalamas et al [53], it is labeled as 1 (see Table S1 in Multimedia Appendix 1).

^b^OR: odds ratio.

^c^Not applicable.

**Table 6:** Logistic regression estimates for variables in model 2 for predicting a pro-environmental tendency^a^ (N=24,156).

Variables			Model 2 (with emotional responses) estimates				
			OR^b^ (95% CI)		*P* value		Pseudo R2
**Meteorological indicators**							
	Extreme hot vs not extreme hot	0.903 (0.822-0.994)		.04		0.356	
**Emotional responses**							
	Emotional well-being	1.415 (1.403-1.427)		<.001		—^c^	
**Cognitive responses**							
	Social affiliations	—		—		—	
	Analytical-intuitive thinking style	—		—		—	
	Somatosensory experiences	—		—		—	
**Regional vulnerability factors**							
	Population density, high vs low	—		—		—	
	Exposure index, high vs low	—		—		—	
	Sensitivity index, high vs low	—		—		—	
	Adaptability index, high vs low	—		—		—	

^a^The pro-environmental tendency is a binary outcome. If a post includes keywords and phrases from any of the seven pro-environmental categories curated and validated by Kalamas et al [53], it is labeled as 1 (see Table S1 in Multimedia Appendix 1).

^b^OR: odds ratio.

^c^Not applicable.

**Table 7:** Logistic regression estimates for variables in model 3 for predicting a pro-environmental tendencya (N=24,156).

Variables		Model 3 (with additional cognitive responses) estimates		
		OR^b^ (95% CI)	*P* value	Pseudo R2
**Meteorological indicators**				
	Extreme hot vs not extreme hot	1.024 (0.922-1.138)	.66	0.413
**Emotional responses**				
	Emotional well-being	1.223 (1.211-1.235)	<.001	—^c^
**Cognitive responses**				
	Social affiliations	1.432 (1.409-1.456)	<.001	—
	Analytical-intuitive thinking style	1.018 (1.015-1.022)	<.001	—
	Somatosensory experiences	1.038 (1.025-1.051)	<.001	—
**Regional vulnerability factors**				
	Population density, high vs low	—	—	—
	Exposure index, high vs low	—	—	—
	Sensitivity index, high vs low	—	—	—
	Adaptability index, high vs low	—	—	—

^a^The pro-environmental tendency is a binary outcome. If a post includes keywords and phrases from any of the seven pro-environmental categories curated and validated by Kalamas et al [53], it is labeled as 1 (see Table S1 in Multimedia Appendix 1).

^b^OR: odds ratio.

^c^Not applicable.

**Table 8:** Logistic regression estimates for variables in model 4 for predicting a pro-environmental tendencya (N=24,156).

Variables			Model 4 (with additional regional vulnerability factors) estimates				
			OR^b^ (95% CI)		*P* value		PseudoR2
**Meteorological indicators**							
	Extreme hot vs not extreme hot	1.010 (0.908-1.122)		.86		0.414	
**Emotional responses**							
	Emotional well-being	1.224 (1.212-1.236)		<.001		—^c^	
**Cognitive responses**							
	Social affiliations	1.433 (1.410-1.456)		<.001		—	
	Analytical-intuitive thinking style	1.019 (1.015-1.022)		<.001		—	
	Somatosensory experiences	1.038 (1.025-1.051)		<.001		—	
**Regional vulnerability factors**							
	Population density, high vs low	0.951 (0.864-1.045)		.30		—	
	Exposure index, high vs low	1.001 (0.906-1.105)		.99		—	
	Sensitivity index, high vs low	0.756 (0.685-0.835)		<.001		—	
	Adaptability index, high vs low	1.254 (1.130-1.393)		<.001		—	

^a^The pro-environmental tendency is a binary outcome. If a post includes keywords and phrases from any of the seven pro-environmental categories curated and validated by Kalamas et al [53], it is labeled as 1 (see Table S1 in Multimedia Appendix 1).

^b^OR: odds ratio.

^c^Not applicable.

## Discussion

### Principal Findings

Climate change is increasingly relevant and concerning due to the ongoing emergence of heat waves and extreme weather events as the new normal [62]. The impact of climate change on individuals extends beyond physical, psychological, cognitive, and behavioral responses. By combining social media data and meteorological data, this study elucidated how extreme meteorological events and changes affected the public’s emotional well-being and the potential pathways of the impacts through three cognitive responses.

First, we found that extreme hot days and hotter temperatures are associated with more negative emotional well-being in climate change discourse. This result was robust after controlling for seasonal and regional vulnerability factors. We also found that extreme cold days are positively associated with expressed emotional well-being, while extreme wet days have a weak association with negative emotional well-being. Although cold weather is often linked to lower moods or increased feelings of isolation, when discussing climate change, the prevailing perception tends to focus on rising temperatures. One possible explanation is that on extremely cold days, people may experience relief from long-standing patterns of increased temperature and express more positive sentiments. In contrast, extreme wet days, characterized by rain and overcast skies, may limit outdoor activities and contribute to feelings of lethargy or negative emotions. However, these two weather indicators do not appear to be associated with the proposed mediators, such as social connections and sensory experiences. Overall, this finding is consistent with previous research that has demonstrated the adverse effects of heat stress on mental health outcomes [4,6,63]. However, in comparison, our chosen outcome of emotional well-being may offer insights into a more immediate and nuanced impact of climate change on individuals’ mood states. Over the long term, if these effects persist and accumulate, they have the potential to contribute to the development of clinical mental disorders. Moreover, the results showed that the impact of extreme hot days on emotional well-being is not uniform across seasons and regions and varies depending on the level of vulnerability to heat risk. Specifically, extreme hot days in the summer and regions with a higher population density are associated with more negative emotional well-being than other seasons and regions. These findings confirm previous evidence that heat waves during summer can cause excessive adverse health consequences [64] and negative sentiments [63], and a high urban land use density (including residential buildings) contributes to increased affective symptoms [65]. Together, these findings suggest that the impact of climate change is not only influenced by objective meteorological conditions but also interacts with environmental (eg, average temperature, air pollution) and social (eg, population density) contexts. Individuals with high-risk exposures and population density or in the summer are more vulnerable to heat, in terms of emotional well-being.

Adding to the existing literature, our study indicates the proposed mediation paths of three cognitive responses of individuals. Specifically, the findings indicate that extreme hot days and hotter temperatures may trigger intuitive-leaning thinking, a less affiliative attitude, and a higher level of somatosensory experience, the former two being associated with more negative emotional well-being. Heat as a stress can comprise people’s cognitive function, which triggers the more cognitive-saving intuitive strategy in thinking [66]. This also confirms previous findings that analytical thinking displays more positive emotions by gaining more control and self-efficacy with rational and logical reasoning [15,67]. Furthermore, the findings are consistent with the literature on social affiliations, which suggests that individuals with high affiliative motives tend to experience positive emotions and exhibit better mental health outcomes [19-21]. Conversely, heat stress, as an immediate and vivid threat, can shift people’s attention to “self” and defensive states and hence make them less socially affiliated. It aligns with previous findings indicating that heat stress can increase individuals’ hostile cognition and aggression [68] and block people’s capacity to care for others (eg, cognitive empathy) [69]. It is noteworthy that although somatosensory experiences were positively associated with extreme heat events, they were unexpectedly found to be positively associated with emotional well-being, contrasting with our initial expectations. This suggests that somatic symptoms induced by heat may not necessarily be linked to more negative emotional well-being. This observation can be supported by the content of several sampled posts with high somatosensory experiences (see Table S2 in Multimedia Appendix 1). A nuanced examination of the post contents found that individuals who shared their somatosensory experiences online may have adapted their observations around their own lives or the internet to make sense of their experiences and proposed environmental protection measures to mitigate the impact. Consequently, expressing somatosensory experiences through social media platforms can serve as a coping method for people dealing with the impacts of climate change and is linked to positive emotional well-being.

We also identified the negative impact of extreme hot days and hotter temperatures on people’s pro-environmental tendencies, which aligns with previous research in highlighting that extreme weather, such as high temperatures and heavy rain, can make people more reluctant to engage in pro-environmental behaviors [70]. However, this effect weakened when controlling for emotional well-being. This indicates that part of the negative association between extreme heat/temperature increase and pro-environmental tendencies may be attributed to the effect of negative emotional well-being. We found that more negative emotional well-being is associated with lower pro-environmental tendencies. This finding contradicts previous studies with coping mechanisms, highlighting that negative emotions increase pro-environmental behaviors as individuals take actions to mitigate the negative impacts caused by climate change [29,71]. This relationship is particularly evident at low-to-moderate levels of climate anxiety, which were found to be associated with increased pro-environmental behavior. However, our results identified an alternative perspective with positive emotional coping strategies. Positive emotional adaptive coping strategies aim to evoke positive feelings to counteract negative emotions [72] and form a positive self-reinforcing feedback loop [73]. This strategy of regulating emotions, also framed as meaning-focused coping, involves individuals adapting their beliefs, values, and goals to maintain well-being [74]. Combining the existing literature with this study suggests a U-shaped correlation between emotional response to climate change and pro-environmental behaviors, rather than a linear one. This finding supports the broader theoretical framework of an inverted U-shaped correlation. The positive emotions arising from meaning-focused coping align with Fredrickson’s broaden-and-build theory [75], which posits that positive emotions can enhance creative problem solving, social cooperation, and resilience—all essential for sustained environmental action. This insight can inform future interventions and theory conceptualization by highlighting that rather than assuming that inducing negative emotions consistently results in increased action, it is also beneficial to leverage positive emotions to establish effective coping strategies and create a self-reinforcing cycle. In our study, we also found that three cognitive strategies, namely engaging in higher analytical thinking, social affiliations to the community, and sharing somatosensory experiences, can help enhance emotional well-being. Adopting these cognitive strategies is also associated with a greater likelihood of advocating pro-environmental behaviors. This is evidenced by several sampled posts that demonstrated co-occurrences between the three cognitive factors and pro-environmental advocacy (Figure S4 in Multimedia Appendix 1). Although the mediation models suggest that cognitive variables would mediate the relationship between extreme weather indices and emotional well-being, it is important to note that the results do not establish a causal relationship. Based on dual process theory [76], it is possible that emotional and cognitive variables mutually influence and jointly contribute to behavioral responses, which can, in turn, foster positive emotions and coping strategies related to climate change. As mentioned earlier in this section, this creates a self-reinforcing positive cycle. Future studies can further explore this relationship in an experimental setting to investigate specific causal relationships.

### Implications

This study has several implications for climate change communication and intervention. First, the findings suggest that social media can be a valuable platform for monitoring and assessing the public’s emotional and cognitive responses to climate change, as well as their pro-environmental tendencies. By analyzing the psycholinguistic cues and contents of social media posts, researchers and practitioners can gain insights into the psychological impact of climate change across populations and regions. The results of this study indicate that future interventions should focus on leveraging positive emotions to develop effective coping strategies. This approach can help create a self-reinforcing cycle in addressing the risks associated with climate change. Second, the findings suggest that affiliative thoughts, more analytic thinking, and expression of somatosensory experiences can be important cognitive strategies to regulate emotional well-being and motivate pro-environmental behaviors of individuals. Climate change communicators and educators can actively use more analytical thinking and affiliative language and encourage sharing personal somatosensory experiences to enhance meaning-focused coping abilities and further enhance the public’s emotional well-being and pro-environmental tendencies. For example, a study examining communication strategies found that environmental groups tend to use more affiliative language, such as emphasizing collective identity and efficacy to engage people in collective action [40]. Third, the findings suggest that climate change communication and intervention should address the regional disparities and vulnerabilities in the impact of climate change to increase the resilience and adaptation of the most vulnerable communities. For instance, in regions characterized by a high population density, a potential strategy is to leverage individuals with high social capital and stronger social networks in the community to enhance adaptation and spread innovations in combating climate change [77].

### Limitations

Despite the potential theoretical and practical contributions of this study, some limitations and directions for future research should be noted. First, this study relied on social media data from Weibo, and hence, the users may not be representative of the general population or users of other social media platforms. Future research could replicate and extend the study on other platforms (eg, X). Second, the study used a correlational design, which does not allow for causal inference or temporal ordering of the variables. Future research could use longitudinal data or experimental designs to establish the causal relationships and mechanisms between meteorological factors, emotional and cognitive responses, and pro-environmental behaviors. Third, due to the data privacy of Weibo, our study did not capture individuals’ sociodemographic data, and hence, these variables were not controlled in the modeling, which may limit the understanding and implications. Fourth, the study focused on a limited set of meteorological factors, emotional and cognitive responses, and pro-environmental behaviors. Future research could explore other meteorological factors, such as wind speed, humidity, and atmospheric pressure, as well as other emotional and cognitive responses, such as hope and moral reasoning, to capture the multifaceted impact of climate change. Although this study offers valuable insights by leveraging data from China—a country notable for its vast geographical diversity and wide range of meteorological and regional vulnerability characteristics—there are inherent limitations regarding the generalizability of the findings to other countries or regions. Differences in climate patterns, topography, socioeconomic factors, and infrastructures worldwide may influence the applicability of the results beyond the Chinese context. Therefore, caution should be exercised when extrapolating these findings to areas with distinct weather conditions or geographic features. Nonetheless, the methodological framework developed in this study provides a robust and adaptable approach that could be effectively applied or modified for similar analyses in other regions, potentially enhancing comparative studies and contributing to a broader understanding of meteorological impacts on emotional well-being worldwide.

### Conclusion

In summary, this study investigated how extreme meteorological events and meteorological changes affect the public’s emotional and cognitive responses to climate change, as well as their pro-environmental tendencies, using social media, meteorological data, and regional vulnerability data. The results showed that extreme hot days and hotter temperatures are robustly associated with more negative emotional well-being, an intuitive-leaning thinking style, a less affiliative attitude, and a lower inclination toward pro-environmental behaviors. The results also revealed regional and seasonal variations in the impact of climate change. Three cognitive responses, namely thinking style, social affiliations, and somatosensory experiences, showed a positive mediated impact on the relationship between climate change-related events and emotional well-being. The findings have implications for climate change communication and intervention, suggesting the need to consider the psychological impact of climate change on different groups and regions and to promote analytical thinking, affiliative language, and positive somatosensory experiences to enhance the public’s mental well-being, coping abilities and pro-environmental behaviors.

## Appendix

Multimedia Appendix 1 Data collection, weather indicators, vulnerability indicators, and cognitive response measurement.

## Abbreviations

AI: artificial intelligence
CFI: comparative fit index
GFI: goodness-of-fit index
LIWC: Linguistic Inquiry and Word Count
OR: odds ratio
PEB: Pro-Environmental Behavior questionnaire
RMSEA: root mean square error of approximation
SC-LIWC: Simplified Chinese version of the Linguistic Inquiry and Word Count
SEM: structural equation modeling
VIF: variance inflation factor

## Data Availability

All data used in this study are publicly available open source data. The details of data collection are reported in the *Data Collection* section in Multimedia Appendix 1.
