# Supplementary material for: Decoding the Public’s Real-Time Emotional and Cognitive Responses to the Changing Climate on Social Media: Computational Analysis Using Weibo and Meteorological Data
Source: J Med Internet Res. 2025 Oct 3;27:e70336. doi: 10.2196/70336 (PMC12534770; doi:10.2196/70336)
Supplement: Multimedia Appendix 1 [file jmir_v27i1e70336_app1.docx]

**Multimedia Appendix 1**

Table of Contents

1. Data collection 2

1.1. Meteorological data 2

1.2. Social media data 2

1.3. Regional vulnerability data 2

2. Weather indicators 2

3. Vulnerability indicators 3

4. Measuring cognitive responses 3

5. Table S1. Pro-environmental keywords and phrases 4

6. Table S2. Descriptive statistics for emotional well-being and cognitive responses 6

7. Table S3. Meteorological indicators across provinces 7

8. Table S4. Regional vulnerability across provinces 7

9. Figure S1. Interaction effect of Season and Temperature changes on emotional well-being 9

10. Figure S2. Interaction effect of population density and temperature changes on emotional well-being 9

11. Figure S3. Mediation paths from SEMs 10

12. Figure S4. Example posts with pro-environmental tendencies in three cognitive dimensions 10

13. Table S5. Sampled posts with somatosensory experiences identified with SC-LIWC 11

# Data collection

## Meteorological data

Daily meteorological data were collected from the Global Historical Climatology Network (GHCN) provided by the National Center for Environmental Information (NOAA) [1]. The NOAA provides daily climate historical data all over the world. We extracted GHCN daily summary data for China from the years 2002 to 2023 by specifying the location as “China” and downloaded data year by year (n(records) = 216,476). We utilized a timeframe of 20 years to ensure that we captured sufficient long-term historical records of meteorological changes that could be used to define extreme weather events and operationalize and evaluate climate change, as recommended by [2]. Although various meteorological data were available, for the current study, we focused on the daily average air temperature and precipitation. Daily average air temperatures were calculated by averaging the maximum daily temperature and minimum daily temperature [3]. We also calculated the daily average air temperature by provinces of China. Since some provinces had multiple weather stations that provided the meteorological data, we took an average of the daily average temperature and precipitation provided by all stations of the province to represent the overall daily meteorological index in that province.

## Social media data

We collected posts mentioning climate change from the most popular social media platform in China—Weibo via Weibo API (Application Programming Interface). Following the method of previous studies on collecting climate change-related posts [4,5], two keywords—“气候变化” (climate change) and “全球变暖” (global warming) were used to retrieve relevant posts from 30 April 2022 to 31 July 2023. The starting date was selected because displaying the IP addresses of users on Weibo has been required by national regulation since that date, which warranted us opportunity to gain a more nuanced and accurate understanding of regional differences in the impact of extreme meteorological events on people’s emotional well-being. The initial screening resulted in a total sample of 76,514 posts. A total of 26,491 posts without IP Addresses were removed. Posts from Taiwan (132), Hong Kong (489), and Macau (55) were also excluded because Weibo is not the predominant social media in these regions. Finally, a total of 42,943 posts were used for analyses, including 24,156 posted by individuals and 18,787 posted by verified accounts. All data were anonymous and privacy was strictly protected during this process, following the privacy and ethical principles listed by [6].

## Regional vulnerability data

Regional-level vulnerability data were collected for the sub-group analysis. Regional-level vulnerability data comprised two parts. The first part included regional vulnerability indexes that were constructed and validated by Zhang et al. (2021). The second part is regional-level population density, which was obtained from China's National Bureau of Statistics [7].

# Weather indicators

An extreme hot day in a region was identified when the average temperature for a day exceeded the 90th percentile of the average temperature for that same date (month-day) over the past 20 years in the same region. Similarly, an extreme cold day was defined when the average temperature for a day was below the 10th percentile of the average temperature for the same date over the past 20 years. The extreme wet day was classified when a day's precipitation in one region exceeded the 90th percentile of the average precipitation for that same date over the past 20 years. The extreme dry day was identified when a day's precipitation in one province fell below the 10th percentile of the average precipitation for that same date over the past 20 years.

To conceptualize and operationalize changing climate, we developed two indicators, namely temperature changes and precipitation changes. These indicators track daily deviations in temperature and precipitation compared to the average values of the same date over the past 20 years. For instance, temperature changes in a particular region on a given day were calculated by subtracting the average temperature recorded on that date over the past 20 years in that region from the temperature of the current day. Similarly, precipitation changes in a region on a specific day were calculated by subtracting the average precipitation recorded on that date over the past 20 years in that region from the precipitation of the current day.

# Vulnerability indicators

Exposure measures the level of individuals’ exposure to hazards that can exacerbate the impact of heat which consists of 6 factors such as frequency of heat waves, PM2.5, and others. Higher exposures are expected to be associated with greater adverse impacts of climate change on emotional well-being. Sensitivity evaluates the degree of impact on a population, considering factors such as the elderly dependency ratio and poverty population ratio. A higher sensitivity level is likely to result in more negative emotional well-being. Adaptability is the ability of a region to cope with heat-related risks, encompassing various factors such as per-capita GDP and green coverage rate. A higher level of adaptability signifies a greater resilience to such risks, which, in turn, is expected to be linked to a more positive emotional well-being. We added population density as the fourth vulnerability indicator which has been identified as a significant indicator of assessing heat vulnerability [8]. It is commonly observed that higher population density is linked to increased vulnerability to heat risk due to heightened human activities and urbanization, which contribute to elevated temperatures and the urban heat island effect.

# Measuring cognitive responses

**Analytical-intuitive thinking style.** Thinking style was measured by several categories in the SC-LIWC dictionary. We constructed a composite index - analytical-intuitive thinking score to represent individuals’ overall thinking style, which was calculated as the score of analytical thinking minus the score of intuitive thinking. The analytical thinking score captures “the degree to which people use words that suggest formal, logical, and hierarchical thinking patterns” [9,10]. It was measured by several categories of keywords and calculated as (articles) + (prepositions) - (total pronouns) - (auxiliary) - (negations) - (conjunctions) - (adverbs) [11]. The intuitive thinking style refers to a more immediate, instinct and intuitive thinking style [12]. The intuitive thinking category includes keywords associated with causation (e.g., “because”), self-reflection (e.g., “feel”), certitude (“really”), and so on. High analytical-intuitive thinking scores indicate an analytical-leaning thinking style while low scores indicate an intuitive-leaning thinking style.

**Social affiliations.** Social affiliation was measured by the category of *affiliation* in the SC-LIWC dictionary. It has been used to capture people’s group identity and connectedness with other group members under the COVID-19 impact [13]. Affiliation keywords capture how people mentioned their friends, groups, and communities when thinking about climate change and extreme heat risks. The keywords include their social connection (e.g., “friend”, “group”, “team”) and collective actions (e.g., help, together). High affiliation scores imply that users recall more of their social connections and collective actions when posting climate change-related posts.

**Somatosensory experience.** Somatosensory experience captures people’s body parts, sensory experience, and perceptual processes (e.g., “feeling”, “heard”, “touch”) when experiencing climate change and heat waves, which are particularly relevant in heat hazards. The Somatosensory experience was measured by the category of perceptual process and body in the SC-LIWC dictionary and was computed as the score of (perceptual processes) plus the score of (body) [11]. High somatosensory scores indicate users evoke more bodily sensations and perceptions when thinking of climate change.

# Table S1. Pro-environmental keywords and phrases

| Chinese version | English version | Category* |
| --- | --- | --- |
| 捐钱 | Donate money | Activism |
| 抵制破坏环境 | Boycott environmental destruction | Activism |
| 倡导环境保护 | Advocate for environmental protection | Activism |
| 签署环境保护请愿书 | Sign an environmental protection petition | Activism |
| 参加志愿活动 | Participate in volunteer activities | Activism |
| 自然保护 | Nature conservation | Activism |
| 环境保护 | Environmental protection | Activism |
| 环保 | Eco-friendly | Activism |
| 买环境安全 | Buy environmentally safe product | Consumption |
| 买有机食品 | Buy organic food | Consumption |
| 可降解包装 | Biodegradable packaging | Consumption |
| 买环保产品 | Buy environmentally friendly products | Consumption |
| 避免使用气溶胶容器 | Avoid aerosol containers | Consumption |
| 不买用动物进行实验的产品 | Do not buy products tested on animals | Consumption |
| 不买来自濒危动物物种的产品 | Do not buy products from endangered species | Consumption |
| 不买对环境造成危害的产品 | Do not buy products that harm the environment | Consumption |
| 不买包装对环境造成危害的产品 | Do not buy products with harmful packaging | Consumption |
| 不买用农药的产品 | Do not buy products with pesticides | Consumption |
| 购买能源效率家用电器 | Buy energy-efficient appliances | Consumption |
| 绿色消费 | Green consumption | Consumption |
| 可持续性消费 | Sustainable consumption | Consumption |
| 减少塑料 | Reduce plastic | Consumption |
| 购买区域性产品 | Buy locally sourced products | Consumption |
| 使用可再生 | Use renewable resources | Consumption |
| 素食 | Vegetarian | Sustainable diet |
| 低碳饮食 | Low-carbon diet | Sustainable diet |
| 植物饮食 | Plant-based diet | Sustainable diet |
| 少吃肉 | Eat less meat | Sustainable diet |
| 回收纸张 | Recycle paper | Recycling |
| 回收纸板 | Recycle cardboard | Recycling |
| 回收塑料瓶 | Recycle plastic bottles | Recycling |
| 回收金属 | Recycle metal | Recycling |
| 回收玻璃瓶 | Recycle glass bottles | Recycling |
| 回收电池 | Recycle batteries | Recycling |
| 可循环利用 | Recyclable | Recycling |
| 可回收包装 | Recyclable packaging | Recycling |
| 随手关灯 | Turn off lights | Saving energy |
| 自然晾干 | Air dry naturally | Saving energy |
| 冬天调低暖气 | Lower heating in winter | Saving energy |
| 用节能灯泡 | Use energy saving bulbs | Saving energy |
| 夏天少用空调 | Use air conditioning less in summer | Saving energy |
| 随手关水龙头 | Turn off taps | Saving energy |
| 节约用电 | Save electricity | Saving energy |
| 节约用水 | Save water | Saving energy |
| 使用可降解 | Use biodegradable products | Saving energy |
| 重复使用 | Reuse | Saving energy |
| 不使用泡沫塑料包装 | Do not use foam plastic packaging | Saving energy |
| 使用清洁能源 | Use clean energy | Saving energy |
| 绿色生活 | Green living | Saving energy |
| 低碳生活 | Low-carbon living | Saving energy |
| 乘坐\|公交车 | Take the bus | Geen transportation |
| 公共交通\|地铁 | Public transportation or subway | Green transportation |
| 火车 | Take train | Green transportation |
| 骑自行车 | Ride a bike | Green transportation |
| 步行 | Walk | Green transportation |
| 拼车 | Share car | Green transportation |
| 减少飞机旅行 | Reduce air travel | Green transportation |
| 垃圾分类 | Waste sorting | Waste reduction |
| 减少浪费 | Reduce waste | Waste reduction |
| 减少排放 | Reduce emissions | Waste reduction |

Note. * The keywords and phrases were primarily based on the PEB questionnaire curated and validated by [14], covering 50 PEB items of 7 categories, including saving energy, consumption, activism, recycling, transportation, diets, and waste management. We translated those item keywords for characterizing PEBs into Chinese before conducting the text matching. We also added several keywords that are commonly used in Chinese culture to characterize sustainable diets, a category missing in the PEB questionnaire [14], for instance, “低碳生活” (low-carbon lifestyle), and “低碳饮食” (low-carbon diet). To validate the effectiveness of the keywords in capturing pro-environmental tendencies, we randomly sampled 200 posts that were labelled as “1” by keywords matching (indicating the existence of pro-environmental tendencies). Two researchers (CX and YY) then independently coded those posts based on the guidelines of the PEB scale and additionally added keywords. A briefing and discussion session has been initiated to reach a consensus before independent coding. The average agreement between the two coders for the classification was 96.3% (98% for YY and 94.5% for CX).

# Table S2. Descriptive statistics for emotional well-being and cognitive responses

| Variables | Scoring method | Mean | SD | Min | 25  Percentile | Median | 75  Percentile | max |
| --- | --- | --- | --- | --- | --- | --- | --- | --- |
| Emotional well-being index | LIWC (positive sentiment score minus negative sentiment scores) | 1.58 | 5.88 | -75.00 | -1.35 | 0.00 | 4.45 | 40.00 |
| Thinking style | LIWC (perceptual process score plus body score) | -28.40 | 14.95 | -128.58 | -37.49 | -25.14 | -20.45 | 44.45 |
| Somatosensory | LIWC (analytical thinking score minus intuitive thinking score) | 3.52 | 3.68 | 0.00 | 0.97 | 2.70 | 5.36 | 50.00 |
| Social affiliations | LIWC (category of affiliation score*)* | 2.25 | 2.93 | 0.00 | 0.00 | 0.97 | 3.75 | 33.33 |
|  |  |  |  |  |  |  |  |  |
|  |  |  | Yes |  | No |  |  |  |
| Pro-environmental tendencies | Binary classification based on occurrence of pro-environmental keywords | | 5694 (23.6%) |  | 18467(76.4%) |  |  |  |

# Table S3. Meteorological indicators across provinces

|  | Daily average temperatures in celsius degree | | | | Daily average precipitation in millimeters | | | |
| --- | --- | --- | --- | --- | --- | --- | --- | --- |
| Provinces (based on IPs) | Mean | SD | Median | Max | Mean | SD | Median | Max |
| Anhui | 17.5 | 9.9 | 17.8 | 33.8 | 3.0 | 7.8 | 0.0 | 71.5 |
| Beijing | 15.8 | 10.8 | 16.9 | 31.9 | 3.2 | 12.4 | 0.0 | 113.3 |
| Fujian | 21.6 | 7.4 | 22.1 | 33.2 | 4.7 | 11.6 | 0.1 | 138.6 |
| Gansu | 9.6 | 10.5 | 9.8 | 26.3 | 0.9 | 1.8 | 0.1 | 15.2 |
| Guangdong | 22.9 | 6.2 | 24.0 | 32.1 | 6.1 | 11.4 | 0.4 | 68.7 |
| Guangxi | 22.8 | 6.7 | 23.8 | 32.6 | 4.3 | 7.5 | 0.7 | 60.7 |
| Guizhou | 16.2 | 7.3 | 17.4 | 27.8 | 2.5 | 5.8 | 0.3 | 48.9 |
| Hainan | 25.7 | 3.6 | 26.3 | 31.2 | 4.4 | 11.0 | 0.3 | 122.0 |
| Hebei | 13.2 | 11.6 | 13.8 | 31.6 | 1.3 | 4.2 | 0.0 | 49.1 |
| Henan | 16.3 | 9.9 | 16.8 | 32.0 | 2.5 | 7.9 | 0.0 | 117.0 |
| Heilongjiang | 3.9 | 15.4 | 6.3 | 26.4 | 1.8 | 3.1 | 0.4 | 22.2 |
| Hubei | 17.9 | 9.2 | 18.6 | 34.1 | 2.8 | 6.4 | 0.1 | 52.6 |
| Hunan | 18.9 | 9.3 | 19.4 | 33.8 | 3.1 | 5.9 | 0.2 | 40.1 |
| Jilin | 7.0 | 13.7 | 8.9 | 27.6 | 1.9 | 4.9 | 0.0 | 43.4 |
| Jiangsu | 16.3 | 10.0 | 16.5 | 33.8 | 2.8 | 7.3 | 0.0 | 58.6 |
| Jiangxi | 20.2 | 9.0 | 20.9 | 34.7 | 4.6 | 9.1 | 0.3 | 73.0 |
| Liaoning | 10.2 | 12.4 | 11.9 | 29.7 | 2.0 | 6.2 | 0.0 | 66.7 |
| Inner Mongolia | 5.4 | 13.9 | 6.9 | 26.5 | 1.3 | 2.2 | 0.2 | 13.8 |
| Ningxia | 10.9 | 11.4 | 11.9 | 29.7 | 0.7 | 2.9 | 0.0 | 31.9 |
| Qinghai | 3.0 | 9.4 | 3.2 | 19.7 | 1.2 | 1.8 | 0.3 | 12.5 |
| Shandong | 14.9 | 10.1 | 15.2 | 31.2 | 2.2 | 6.6 | 0.0 | 54.2 |
| Shanxi | 12.0 | 11.0 | 12.7 | 30.1 | 1.4 | 3.9 | 0.0 | 30.9 |
| Shaanxi | 12.8 | 10.1 | 13.6 | 29.4 | 1.9 | 4.9 | 0.0 | 37.3 |
| Shanghai | 18.0 | 9.5 | 18.3 | 34.7 | 3.3 | 9.9 | 0.0 | 93.0 |
| Sichuan | 13.3 | 6.8 | 14.0 | 26.3 | 2.4 | 3.3 | 1.1 | 22.9 |
| Tianjin | 14.3 | 11.4 | 15.0 | 32.5 | 2.0 | 9.6 | 0.0 | 121.4 |
| Tibet | 5.0 | 8.1 | 5.2 | 18.1 | 1.2 | 2.1 | 0.3 | 14.0 |
| Xinjiang | 11.1 | 13.3 | 12.7 | 30.8 | 0.9 | 2.3 | 0.1 | 17.8 |
| Yunnan | 17.5 | 4.5 | 18.4 | 25.2 | 2.6 | 4.1 | 0.7 | 24.6 |
| Zhejiang | 19.1 | 8.5 | 19.1 | 34.2 | 4.0 | 7.7 | 0.3 | 76.8 |
| Chongqing | 16.5 | 8.8 | 17.2 | 31.9 | 3.5 | 12.9 | 0.0 | 244.6 |

# Table S4. Regional vulnerability across provinces

| Provinces | Exposure index score | Sensitivity index score | Adaptability index score | Vulnerability index score | Population per square kilometer |
| --- | --- | --- | --- | --- | --- |
| Anhui | 0.122 | −0.011 | 0.14 | −0.029 | 2744 |
| Beijing | 0.118 | 0.012 | 0.231 | −0.100 | 1331 |
| Fujian | 0.073 | 0.075 | 0.179 | −0.032 | 3492 |
| Gansu | 0.068 | −0.005 | 0.049 | 0.014 | 3223 |
| Guangdong | 0.089 | 0.121 | 0.23 | −0.020 | 3856 |
| Guangxi | 0.075 | 0.041 | 0.143 | −0.028 | 2473 |
| Guizhou | 0.063 | −0.006 | 0.09 | −0.033 | 2092 |
| Hainan | 0.037 | 0.109 | 0.142 | 0.004 | 2487 |
| Hebei | 0.148 | −0.017 | 0.127 | 0.004 | 3150 |
| Henan | 0.154 | 0.004 | 0.123 | 0.035 | 4480 |
| Heilongjiang | 0.135 | −0.023 | 0.161 | −0.049 | 5361 |
| Hubei | 0.109 | 0.006 | 0.13 | −0.015 | 3056 |
| Hunan | 0.103 | 0.031 | 0.141 | −0.006 | 4717 |
| Jilin | 0.049 | −0.026 | 0.074 | −0.051 | 2097 |
| Jiangsu | 0.112 | −0.013 | 0.231 | −0.132 | 2156 |
| Jiangxi | 0.097 | 0.008 | 0.142 | −0.036 | 3647 |
| Liaoning | 0.08 | −0.004 | 0.122 | −0.047 | 1792 |
| Inner Mongolia | 0.048 | −0.023 | 0.114 | −0.090 | 2002 |
| Ningxia | 0.083 | −0.014 | 0.1 | −0.031 | 3103 |
| Qinghai | 0.034 | 0.123 | 0.046 | 0.112 | 2893 |
| Shandong | 0.111 | −0.030 | 0.163 | −0.082 | 1724 |
| Shanxi | 0.133 | 0.021 | 0.107 | 0.047 | 3855 |
| Shaanxi | 0.139 | 0.001 | 0.104 | 0.036 | 5321 |
| Shanghai | 0.135 | −0.023 | 0.161 | −0.049 | 3905 |
| Sichuan | 0.097 | −0.048 | 0.146 | −0.097 | 3670 |
| Tianjin | 0.157 | 0.015 | 0.096 | 0.076 | 4372 |
| Tibet | 0.016 | 0.262 | 0.096 | 0.182 | 1516 |
| Xinjiang | 0.091 | 0.096 | 0.112 | 0.075 | 3915 |
| Yunnan | 0.036 | 0.02 | 0.088 | −0.032 | 3290 |
| Zhejiang | 0.112 | 0.007 | 0.198 | −0.079 | 2344 |
| Chongqing | 0.158 | −0.006 | 0.128 | 0.024 | 2079 |

Note. Regional vulnerability indexes that were constructed and validated by Zhang et al [15]. Population density was obtained from China's National Bureau of Statistics [7].

# Figure S1. Interaction effect of Season and Temperature changes on emotional well-being


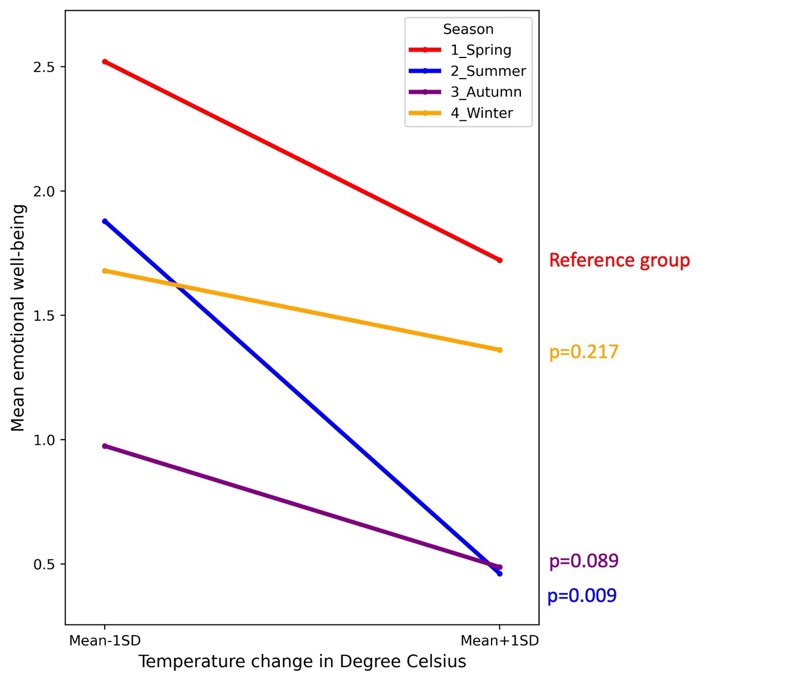


Seasons are categorized by a three-month interval where December to February is winter, March to May is spring, June to August is summer, and September to November is autumn. X-axis represents the temperature change in Degree Celsius. The left side represents the temperature change to colder (Mean-1SD) and the right side represents the temperature change to hotter (Mean+1SD). The Y-axis represents the mean score of emotional well-being.

# Figure S2. Interaction effect of population density and temperature changes on emotional well-being


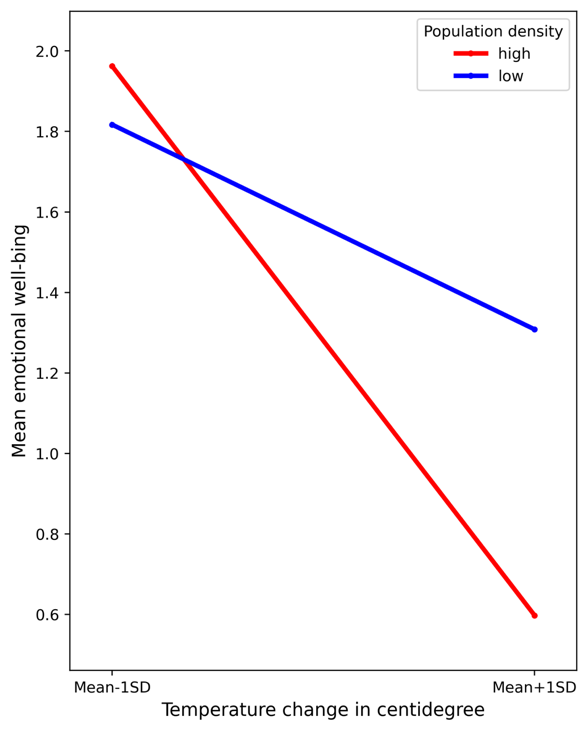


High population density indicates that the region’s population density is higher than the median of all provinces. X-axis represents the temperature change in Degree Celsius. The left side represents the temperature change to colder (Mean-1SD) and the right side represents the temperature change to hotter (Mean+1SD). The Y-axis represents the mean emotional well-being.

# Figure S3. Mediation paths from SEMs


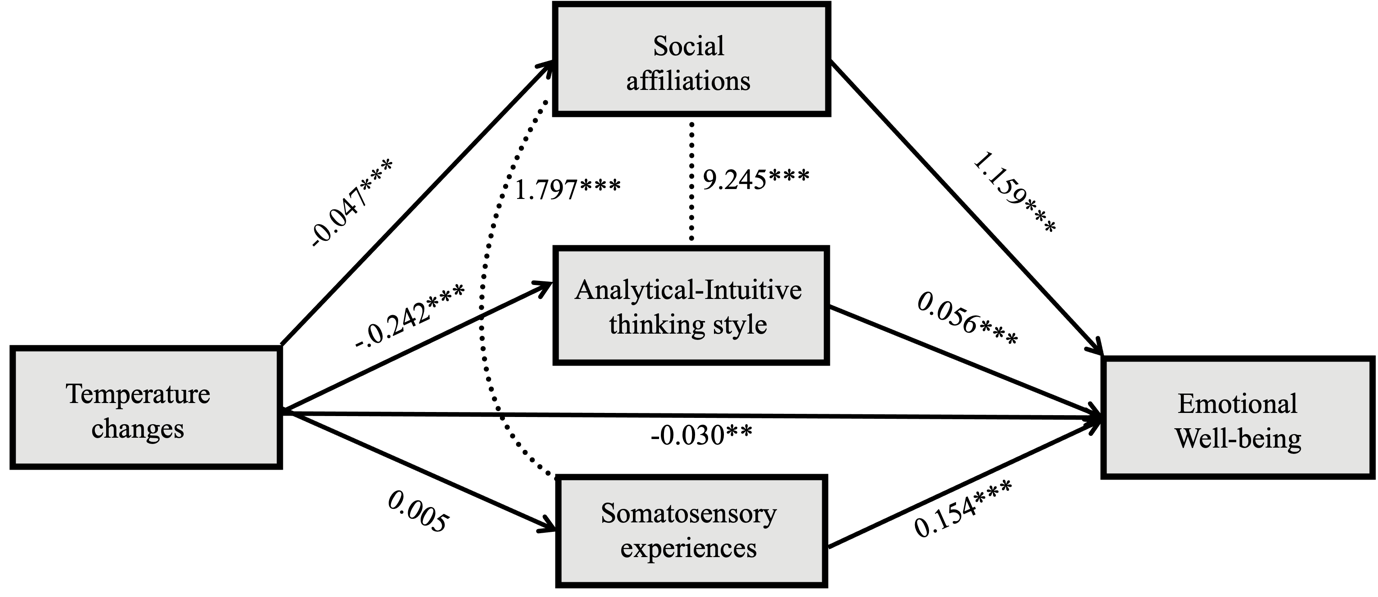


CFI 0.996, GFI 0.999, rmsea, 0.047

# Figure S4. Example posts with pro-environmental tendencies in three cognitive dimensions


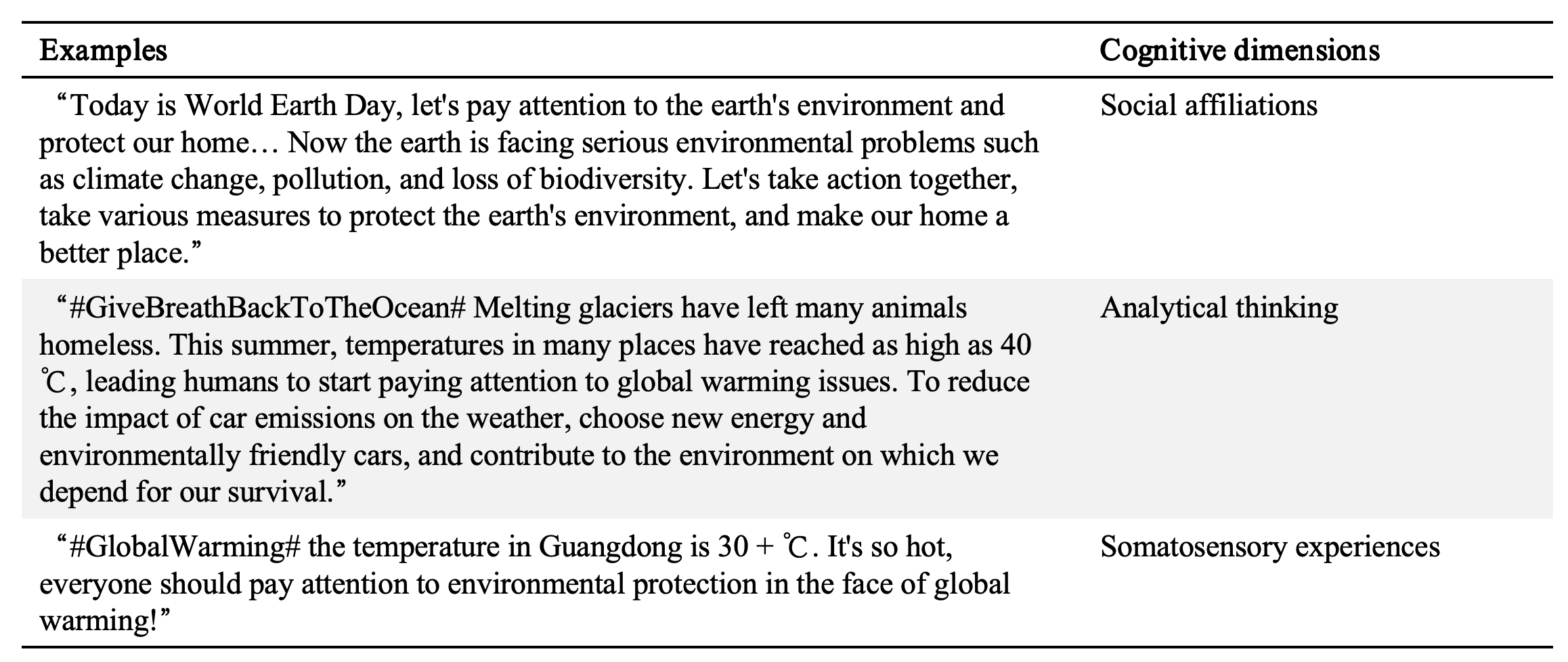


# Table S5. Sampled posts with somatosensory experiences identified with SC-LIWC

| Original posts | English version | Topics |
| --- | --- | --- |
| "#全国多地出现囤冰箱空调热潮# 确实是，感觉随着全球变暖，现在各地区气温都普遍升高了，制冷电梯也用的越来越多。 | #National trend of stockpiling refrigerators and air conditioners emerges# Indeed, I am feeling that with global warming, temperatures in various regions are generally rising, and the use of air conditioning and refrigeration is increasing. | Making sense of their experiences. |
| #全球热#广东30+ 我尊滴好热好热 全球变暖大家要注意环境保护啊 | #Global heat# Guangdong's temperature is over 30 degrees, it's so hot! Everyone should pay attention to environmental protection in the face of global warming. | Pro-environment advocation |
| #全球变暖#真的感觉好明显 真的应该保护环境 | #GlobalWarming# It really feels obvious. We should definitely protect the environment. | Pro-environment advocation |

## References

1. National Oceanic and Atmospheric Administration. National Centers for Environmental Information. 2023 [cited 2023 September 15]; Available from: https://www.ncei.noaa.gov/.
2. VijayaVenkataRaman S, Iniyan S, Goic R. A review of climate change, mitigation and adaptation. Renew Sust Energ Rev. 2012 Jan;16(1):878-97. PMID: WOS:000298764500079. doi: 10.1016/j.rser.2011.09.009.
3. WMO. Guide to Climatological Practices WMO-No. 100. 2018.
4. Pearce W, Niederer S, Özkula SM, Querubín NS. The social media life of climate change: Platforms, publics, and future imaginaries. Wires Clim Change. 2019 Mar-Apr;10(2). PMID: WOS:000458907600005. doi: 10.1002/wcc.569.
5. Schuldt JP, Konrath SH, Schwarz N. “Global warming” or “climate change”? Whether the planet is warming depends on question wording. Public opinion quarterly. 2011;75(1):115-24. doi: 10.1093/poq/nfq073.
6. Kosinski M, Matz SC, Gosling SD, Popov V, Stillwell D. Facebook as a Research Tool for the Social Sciences. Am Psychol. 2015 Sep;70(6):543-56. PMID: WOS:000361041400005. doi: 10.1037/a0039210.
7. National Bureau of Statistics. National Data. 2023 [cited 2024 January 10]; Available from: https://data.stats.gov.cn/.
8. Hanberry BB. Global population densities, climate change, and the maximum monthly temperature threshold as a potential tipping point for high urban densities. Ecol Indic. 2022 Feb;135. PMID: WOS:000761380500002. doi: 10.1016/j.ecolind.2021.108512.
9. Pennebaker JW, Chung CK, Frazee J, Lavergne GM, Beaver DI. When Small Words Foretell Academic Success: The Case of College Admissions Essays. Plos One. 2014 Dec 31;9(12). PMID: WOS:000347119100070. doi: 10.1371/journal.pone.0115844.
10. Jordan KN, Sterling J, Pennebaker JW, Boyd RL. Examining long-term trends in politics and culture through language of political leaders and cultural institutions (vol 116, pg 3476, 2019). P Natl Acad Sci USA. 2019 Apr 2;116(14):7148-. PMID: WOS:000463069900099. doi: 10.1073/pnas.1903863116.
11. Monzani D, Vergani L, Pizzoli SFM, Marton G, Pravettoni G. Emotional Tone, Analytical Thinking, and Somatosensory Processes of a Sample of Italian Tweets During the First Phases of the COVID-19 Pandemic: Observational Study. Journal of Medical Internet Research. 2021 Oct 27;23(10). PMID: WOS:000715045200001. doi: 10.2196/29820.
12. Ashokkumar A, Pennebaker JW. Social media conversations reveal large psychological shifts caused by COVID-19's onset across U.S. cities. Sci Adv. 2021 Sep 24;7(39):eabg7843. PMID: 34550738. doi: 10.1126/sciadv.abg7843.
13. Ashokkumar A, Pennebaker JW. Tracking group identity through natural language within groups. Pnas Nexus. 2022 May;1(2). PMID: WOS:001063384200001. doi: 10.1093/pnasnexus/pgac022.
14. Kalamas M, Cleveland M, Laroche M. Pro-environmental behaviors for thee but not for me: Green giants, green Gods, and external environmental locus of control. J Bus Res. 2014;67(2):12-22. doi: 10.1016/j.jbusres.2013.03.007.
15. Zhang X, Li Y, Cheng Y, Wang Y, Wang Y, Yao X. Assessment of Regional Health Vulnerability to Extreme Heat - China, 2019. China CDC Wkly. 2021 Jun 4;3(23):490-4. PMID: 34594920. doi: 10.46234/ccdcw2021.126.
